# Editorial for the special issue "Olfactory Coding and Circuitries"

**DOI:** 10.1007/s00441-020-03389-1

**Published:** 2021-01-15

**Authors:** Silke Sachse, Ivan Manzini

**Affiliations:** 1 Department of Evolutionary Neuroethology, Max Planck Institute for Chemical Ecology, 07745 Jena, Germany; 2 Department of Animal Physiology and Molecular Biomedicine, Justus-Liebig-University Giessen, Giessen, Germany

**Fig. 1:**
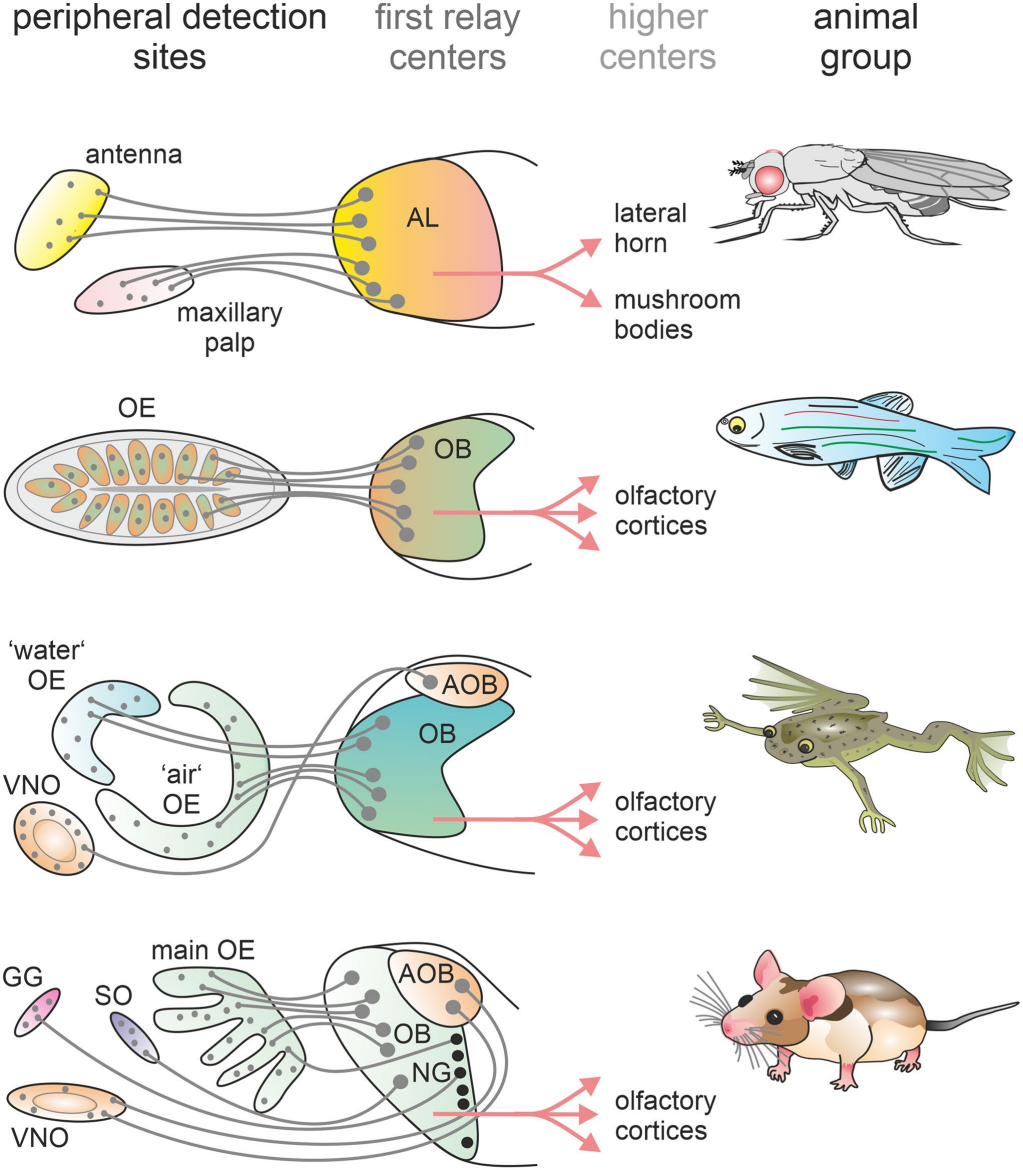
**Organization of the olfactory system in different animal groups** The schematics depict the general organization of olfactory systems of insects (upper panel), fishes (upper middle panel), frogs (lower middle panel), and rodents (lower panel). The schemes are simplified and show only one side of the olfactory system. In insects, olfactory receptor neurons (small grey dots) reside in the antennae and the maxillary palps and project their axons to glomeruli (large grey dots) in the antennal lobe (AL), the first olfactory relay center in insects. Axons of projection neurons in the AL convey the olfactory information to higher olfactory centers (mushroom body and lateral horn). The olfactory systems of fishes consist of a single olfactory epithelium (OE) and a single olfactory bulb (OB). Receptor neurons in the periphery transmit the olfactory information to glomeruli in the OB and from there to higher brain centers via projection neurons. Frogs and rodents have anatomically segregated olfactory subsystems. The clawed frog has a dual main olfactory system consisting of two peripheral epithelia associated with aquatic and aerial olfaction and a vomeronasal organ. Receptor neurons from the two main OE project their axons to different parts of the main OB. Receptor neurons of the vomeronasal organ project to the accessory OB. Projection neurons of both the main- and accessory OB send axons to various higher brain centers. In rodents, in addition to the main- and accessory olfactory system, there are two more anatomically segregated olfactory subsystems, the Grueneberg ganglion and the Septal organ. Receptor neurons in the peripheral epithelia project their axons into different main- and accessory OB zones. From there, different classes of projection neurons transmit the olfactory information to several higher olfactory brain centers **Abbreviations:** AL, antennal lobe; OE, olfactory epithelium; OB, olfactory bulb; AOB, accessory olfactory bulb; VNO, vomeronasal organ; GG, Grueneberg ganglion; SO, Septal organ; NG, necklace glomeruli (black dots)

Olfaction represents one of the most ancient sensory systems. Almost all organisms of divergent animal phyla are equipped with an olfactory system that enables them to detect, encode and interpret odor molecules in their environment. Notably, the world of odors represents a giant, multidimensional and diverse stimulus space that cannot be classified along any narrow set of dimensions. Hence, the nervous system has accomplished a highly challenging task by encoding this complex stimulus space into a meaningful neuronal representation in the brain. In the last decades – and particular very recent years – excellent progress has been made that significantly advanced our understanding about the neurobiological mechanisms that give rise to olfactory percepts. Multiple studies in numerous animal species have shed light on the different stages of the olfactory code, the neurons involved and the transition along the olfactory pathway, i.e., from the peripheral olfactory organs (nose or antennae/maxillary palps) over the first olfactory center in the brain to the higher processing areas which subsequently facilitate odor-guided decisions and behavior. In Fig. 1 we give a schematic overview of the different stages of the olfactory systems of insects, fishes, amphibia, and rodents.

This special issue attempts to summarize our current knowledge on the odor processing mechanisms and its underlying neuronal circuitry in various animal species covering the invertebrate’s as well as vertebrate’s olfactory system. With this review series we would like to emphasize the importance of addressing biological questions in comparative and evolutionary terms in order to elucidate the different strategies that have been evolved to encode the giant world of odors. All animals are faced with the same difficult task to detect the vast array of diverse odor molecules in the environment and to translate that information into an accurate neuronal map resulting in an appropriate behavioral output. In addition, we would like to give the readers a comprehensive insight into the fascinating capacity of the brain to decode the enormous and complex odor space. Thereby, some reviews specifically focus on the adaptations of the olfactory systems that were necessary for a transition from aquatic to aerial olfaction.

In the first part of this special series we aim at reviewing comprehensively the principles of odor coding in various invertebrate species ranging from cockroaches, moths and honeybees to flies and mosquitos, while the second part is dedicated to the olfactory circuitry of vertebrates. We start our review series with summarizing what is known about peripheral odor coding in insects and the involved olfactory receptors, while we proceed with describing the coding principles in the first, second and higher order insect brain centers. Next, we shed light on temporal aspects of the odor stimulus and sensory adaptation. Finally, we focus on the modulation of olfactory processing in the insect brain by internal state, learning or previous experience.

The first review gives insights into the latest view on the molecular and functional properties of olfactory receptors that are expressed in the olfactory organs of insects, the antennae and – additionally in dipterans – the maxillary palps. Two protein families have been identified to be involved in insect olfaction, the ionotropic receptors and the odorant receptors, both of which are proposed to act as ligand gated ion channels, while odorant receptors also function as metabotropic receptors. The review describes in detail the evolution, function and regulation of these two receptor families by focusing mainly on studies that were conducted in the vinegar fly *Drosophila melanogaster* (Wicher and Miazzi 2021). In addition to olfactory receptors, other proteins are expressed in the insect antennae that orchestrate and facilitate the odor detection process. The so-called sensory neuron membrane proteins (SNMPs) are the topic of the review by Cassau and Krieger (2021) that reports the latest advances regarding the functional impact of SNMPs in the processing of olfactory information. The authors describe the discovery and typology of these membrane proteins, as well as their distribution in neuronal and non-neuronal structures by focusing on studies obtained in various insect species, such as moths, flies and locusts.

We next proceed to shed light on the odor coding properties of neuronal circuits in the insect brain. The review by Paoli and Galizia (2021) covers a wide range of processing levels involved in olfactory coding in the honeybee brain by taking into account the many facets of research done in this social insect. Odor coding in the cockroach antennal lobe (AL) – the analogues structure to the vertebrate’s olfactory bulb (OB) – is the topic of the review by Fusca and Kloppenburg (2021) who summarize key physiological properties of the main neurons in the AL, which are the projection neurons and local interneurons. Wheelwright et al. (2021) give a comprehensive and up-to-date overview on odor coding in mosquitoes and highlight particular cases from less studied and obscure mosquito species. Most importantly the authors also emphasize in their review outstanding questions and knowledge gaps in the field of mosquito chemosensation and chemical ecology.

We next turn our attention to olfactory circuits in higher order olfactory centers. Puñal et al. (2021) provide a complete and comprehensive overview of the mushroom body (MB) which represents a quite conserved structure among various arthropod species and is considered to be the key structure for associative learning and memory. The review focusses on the mechanisms underlying the MB development, its function and cellular composition, as well as possible parallels that can be drawn with the development of the mammalian cerebellum. The second higher order brain center that has recently gained increasing attention is the lateral horn (LH), which has emerged as the center of integrating innate behavior and shares similarities with the cortical amygdala in mammals. By focusing on mainly recently published studies, Das Chakraborty and Sachse (2021) summarize the current knowledge of the neuronal circuitry in the LH with regard to odor tuning properties, neurotransmitter identities as well as implications related to odor-guided behavior.

While most studies on olfactory research are conducted with a short, but static odor stimulus system, the temporal structure of an odor plume is often neglected. However, temporal aspects are also neuronally encoded and are shown to affect olfactory detection subsequently. Temporal processing and adaptation in the olfactory system is addressed in the review by Brandão et al. (2021) who describe the information flow at the various stages of processing in *Drosophila* and how it is altered by odor stimulation history from an experimental as well as theoretical perspective. Jafari and Alenius (2021) complement this topic by providing insight into the complex processes underlying odor adaptation in the peripheral and central olfactory system in the fly including its molecular players.

The last part of the invertebrate review series focusses on modulation and plasticity along the olfactory pathway. Anton and Rössler (2021) cover structural and physiological neural plasticity and modulation of olfactory circuits that influence insect behavior and ecology. The authors put particular emphasis on studies performed in moths and social hymenopterans and provide an exhaustive panorama of the current knowledge with regard to the species-specific behavioral and ecological context. Marachlian et al. (2021) review the role of learning in odor recognition and discrimination in the AL of the honeybee by summarizing functional, behavioral and pharmacological studies. The review of Mariette et al. (2021) is also focused on the olfactory system of honeybees, but addresses the cellular and neural mechanisms underlying mating behavior and sex pheromone communication with the special emphasis on the interaction between drones and queens. Also odor detection in mosquitoes is strongly modulated by life stage and internal state. Hill and Ignell (2021) comprehensively review the current knowledge of the mosquito olfactory system with the focus on the plasticity of mosquito behavior and its physiological and molecular bases. The last review of the invertebrate section by Siju et al. (2021) describes modulation in *Drosophila*, by focusing on the role of the neuromodulator dopamine in olfactory processing and behavior. The authors review in detail how the dopaminergic system integrates external and internal cues, and place the findings in the fly in the context of how it works in mammals.

In the second part of this special issue, we attempted to give a comprehensive overview of various aspects of the vertebrate olfactory system. Thereby, we aimed to cover several vertebrate classes, including humans.

The first series of reviews about vertebrates focusses on the olfactory systems of aquatic and amphibious species, deals with the adaptations necessary for a transition from aquatic to aerial olfaction, and discusses the difficulties that secondarily aquatic vertebrates had to readjust the olfactory system to an aquatic habitat. The first review gives a comprehensive overview of the anatomy of the olfactory structures in zebrafish and summarizes the current knowledge of constitutive and regenerative neurogenesis at several levels of the olfactory system (Calvo-Ochoa et al. 2021). A comparison to mammalian olfactory neurogenesis reveals similarities and several differences between species. The contribution by Braubach and Croll (2021) focusses on the glomerular network of the zebrafish OB. In particular, the authors systematically investigated the relationships between distinct bulbar projection neuron types with differently sized glomeruli and discuss the possible implications of their findings. Gerlach and Wullimann (2021) describe the neural pathways of olfactory kin imprinting and kin recognition in zebrafish and discuss the underlying neuronal networks and olfactory centers in a comparative context. The review by Jungblut et al. (2021) starts a series of three contributions dealing with olfaction in amphibians, a class of vertebrates living at the interface between aquatic and terrestrial habitats. Their article gives an extensive overview of the anatomy and the olfactory receptors of the peripheral olfactory subsystems of larval and adult anuran amphibians. The challenge of the anuran olfactory system to function in water and on land is addressed in the contribution of Weiss et al. (2021). These authors also provide a detailed overview of the anuran olfactory structures on a tissue, cellular and molecular level and review the differences and similarities between the olfactory systems of anurans and other vertebrates. A short overview of the olfactory system of urodele amphibians and an extensive review of their pheromonal communication is provided by Woodley and Staub (2021). The last two contributions of the first series of articles deal with the olfactory systems of secondarily aquatic vertebrates species, that faced the challenge to readapt to a life in water. We will learn that this readaptation was often not very successful. The article by Kondoh et al. (2021) is about sea turtles and how they readapted their olfactory system to an aquatic environment and seawater flow. Kishida (2021) gives a broader overview of the olfactory adaptations in aquatic amniotes, including cetaceans, sea snakes, sirenians, pinnipeds, and otters.

The second series of reviews focusses on different families of olfactory receptors and their behavioral relevance, sheds light on the functional relevance of the olfactory marker protein, and comprehensively elucidates the importance of the cAMP signaling pathway in the vertebrate main olfactory system. The first paper of this series extensively reviews the coding of pheromones by the different olfactory receptor families expressed in the vomeronasal organ (Tirindelli 2021). Boillat et al. (2021), on the other hand, review in detail the expression and function of formyl peptide receptors in the immune system and the vomeronasal organ. Thereby, they particularly focus on the neofunctionalization of these receptors from immune pathogen sensors to olfactory chemosensors. The current knowledge on the olfactory trace amine-associated receptors is comprehensively reviewed in the contribution of Dewan (2021). This review also discusses whether trace amine associated receptors represent a unique subsystem within the main olfactory system. The paper by Dibattista et al. (2021) discusses the functional relevance of the olfactory marker protein, a small cytoplasmic protein that marks mature receptor neurons. Its function is still not completely clear, and recent findings that it is expressed also in extra nasal tissues are additionally intriguing. In the last paper of this series, Boccaccio et al. (2021) comprehensively review the different functions of the cyclic AMP signaling pathway in the rodent main olfactory system. As this signaling pathway is also present in receptor neurons of several other vertebrate species, this review is certainly of interest beyond the rodent system.

The third series of reviews mainly focusses on the different stages of the rodent olfactory system, i.e., the olfactory epithelium (OE), the OB, and higher olfactory centers. Also, some smaller rodent olfactory subsystems are introduced and reviewed. The contribution of Kurian et al. (2021) reviews the current knowledge of how odors are coded in the OE, the first stage of the olfactory system. This review deals with the mechanism of combinatorial receptor coding and provides a possible explanation as to why different odorants evoke a distinct percept. Furthermore, it elucidates that antagonistic interactions also play an essential role in forming the combinatorial receptor code. The still non completely answered question of how a precise spatial arrangement of glomeruli in the OB emerges from a random distribution of receptor neurons in the OE is tackled in the contribution of Lodovichi (2021). Marin et al. (2021), on the other hand, deal with the question of how odor information can be sampled and how it might be represented and processed by the mammalian olfactory system. Thereby, these authors mainly focus on how mammals extract spatial information from the environment. Recent findings describing how the hedonic value of an odorant, i.e., whether an odorant is perceived as pleasant or unpleasant, is represented in the OB circuitry are reviewed by Kermen et al. (2021). The next review deals with the role of granule cells, the most prominent interneurons of the OB. Egger and Kuner (2021) summarize recent results about gamma-aminobutyric acid (GABA) release mechanisms and the behavioral relevance of granule cell activity during odor discrimination. The activity of the bulbar neuronal network is influenced by many factors, either from within or outside the OB. As the OB receives massive efferent input from several higher brain centers, extrinsic neuromodulation is likely of fundamental importance. In their contribution, Brunert and Rothermel (2021) review the current knowledge about extrinsic neuromodulatory processes. Thereby, they particularly focus on the origin of extrinsic neuromodulators, the involved receptors, the affected bulbar circuits, and the resulting changes in behavior. The knowledge about how chemical signals are processed in higher brain centers is reviewed by Mucignat-Caretta (2021). She focusses particularly on intraspecific chemical signals and discusses recent findings that support the view that intraspecific signals may activate both the main and the accessory olfactory system. The last two contributions of this series review the current knowledge about the Grueneberg ganglion, a small olfactory subsystem that is involved in the detection of olfactory and thermosensory signals (Fleischer 2021), and explore the olfactory subsystems associated with the characteristic necklace glomeruli in the OB (Zimmermann and Munger 2021).

Finally, the fourth and last series of reviews about vertebrate olfactory systems puts the focus on the significance of olfaction in humans. Boesveldt and Parma (2021) discuss the importance of the olfactory system in human well-being through nutrition and social behavior. The causes and consequences of a disruption of the human sense of smell and currently available treatments of olfactory dysfunction are reviewed by Schäfer et al. (2021). The last review of this series deals with genetic influence of autism candidate genes on circuit wiring and olfactory decoding (Hartig et al. 2021). This review in detail examines the relationship between social odor processing in humans and rodents in the context of health and autism spectrum disorder.

Taken together, we believe that this special issue provides an up-to-date and comprehensive overview of the olfactory system in a variety of species across phyla. While not all-encompassing, to the best of our knowledge, this collection is one of the most extensive of its kind. The reviews cover the various stages of the olfactory system and summarize the current knowledge about the peripheral detection of odorants, their processing in the first relay centers of the olfactory system, i.e., the OB in vertebrates and the AL in insects, and their terminal processing in higher olfactory centers. We hope that this article collection will be beneficial to our colleagues in the field of olfaction and especially to students that are at the very beginning of their journey through this beautiful sense. We truly hope that the reviews will stimulate the readership to elaborate on new ideas and research concepts that will advance the knowledge about the olfactory system in future studies.

We want to thank all the authors who have provided reviews about their special area of expertise and contributed to this special issue. A special thank also goes to the many anonymous referees who sacrificed their valuable time and provided constructive suggestions that significantly increased the quality of the special issue. A special thank goes to Dr. Lukas Weiss for generating single neuron stainings of the OB of *Xenopus* tadpoles and designing the cover of the special issue and Benjamin Fabian for generating single neuron stainings of the fly AL.

Finally, we would like to express our gratitude to the editor in chief, Klaus Unsicker, who initiated this special issue and invited us to put together this collection. A great thank you also goes to Jutta Jäger. All would not have been possible without her continuous and excellent editorial assistance.
